# Generalism drives abundance: A computational causal discovery approach

**DOI:** 10.1371/journal.pcbi.1010302

**Published:** 2022-09-29

**Authors:** Chuliang Song, Benno I. Simmons, Marie-Josée Fortin, Andrew Gonzalez

**Affiliations:** 1 Department of Biology, Quebec Centre for Biodiversity Science, McGill University, Montreal, Canada; 2 Department of Ecology and Evolutionary Biology, University of Toronto, Toronto, Canada; 3 Department of Ecology and Conservation, University of Exeter, Cornwall Campus, Penryn, United Kingdom; University of Chicago, UNITED STATES

## Abstract

A ubiquitous pattern in ecological systems is that more abundant species tend to be more generalist; that is, they interact with more species or can occur in wider range of habitats. However, there is no consensus on whether generalism drives abundance (a selection process) or abundance drives generalism (a drift process). As it is difficult to conduct direct experiments to solve this chicken-and-egg dilemma, previous studies have used a causal discovery method based on formal logic and have found that abundance drives generalism. Here, we refine this method by correcting its bias regarding skewed distributions, and employ two other independent causal discovery methods based on nonparametric regression and on information theory, respectively. Contrary to previous work, all three independent methods strongly indicate that generalism drives abundance when applied to datasets on plant-hummingbird communities and reef fishes. Furthermore, we find that selection processes are more important than drift processes in structuring multispecies systems when the environment is variable. Our results showcase the power of the computational causal discovery approach to aid ecological research.

## Author summary

Ever since Aristotle, the chicken-or-egg causality dilemma has baffled researchers. Such causality dilemmas are abundant in ecological research, where causal directions are often assumed but not tested. An archetypal example is whether being a generalist causes a species to be more abundant, or whether being more abundant causes a species to be generalists. Without doubt, the gold standard to establish causal directions is controlled experiments. However, controlled experiments that can disentangle the direction of causality in this case are challenging because it involves controlling biotic or abiotic niche breadth. These challenges create an opportunity for computational tools to detect the most likely causal direction. Here, by adapting a set of recently developed computational methods, we provide strong evidence that generalism drives abundance, overturning the previously established direction. We hope our work raises awareness of the potential for computational discovery methods to address long-standing questions in ecology, especially increasingly large datasets become available.

## Introduction

Identifying the causes of species abundance is a central question in ecology with direct implications for conservation management [1–3]. A ubiquitous ecological pattern in ecological communities is the skewed distribution of abundance with a few abundant species accompanied by many less abundant and rare species [4]. What causes this species abundance distribution is one of the most studied questions in ecological research. Theoretical studies have provided a diverse array of explanations for the emergence of uneven species abundance distributions, such as neutral theory [5, 6], niche partitioning [7–10], emergent neutrality [11, 12], and even statistical artifacts [13–15]. However, while these well-established theoretical explanations operate under different mechanisms, they generate similar and often empirically indistinguishable patterns (e.g., log-normal distributions). Thus, by considering only species abundance distributions, it is difficult to discern the main ecological drivers of this ecological pattern [16–18].

**Fig 1:**
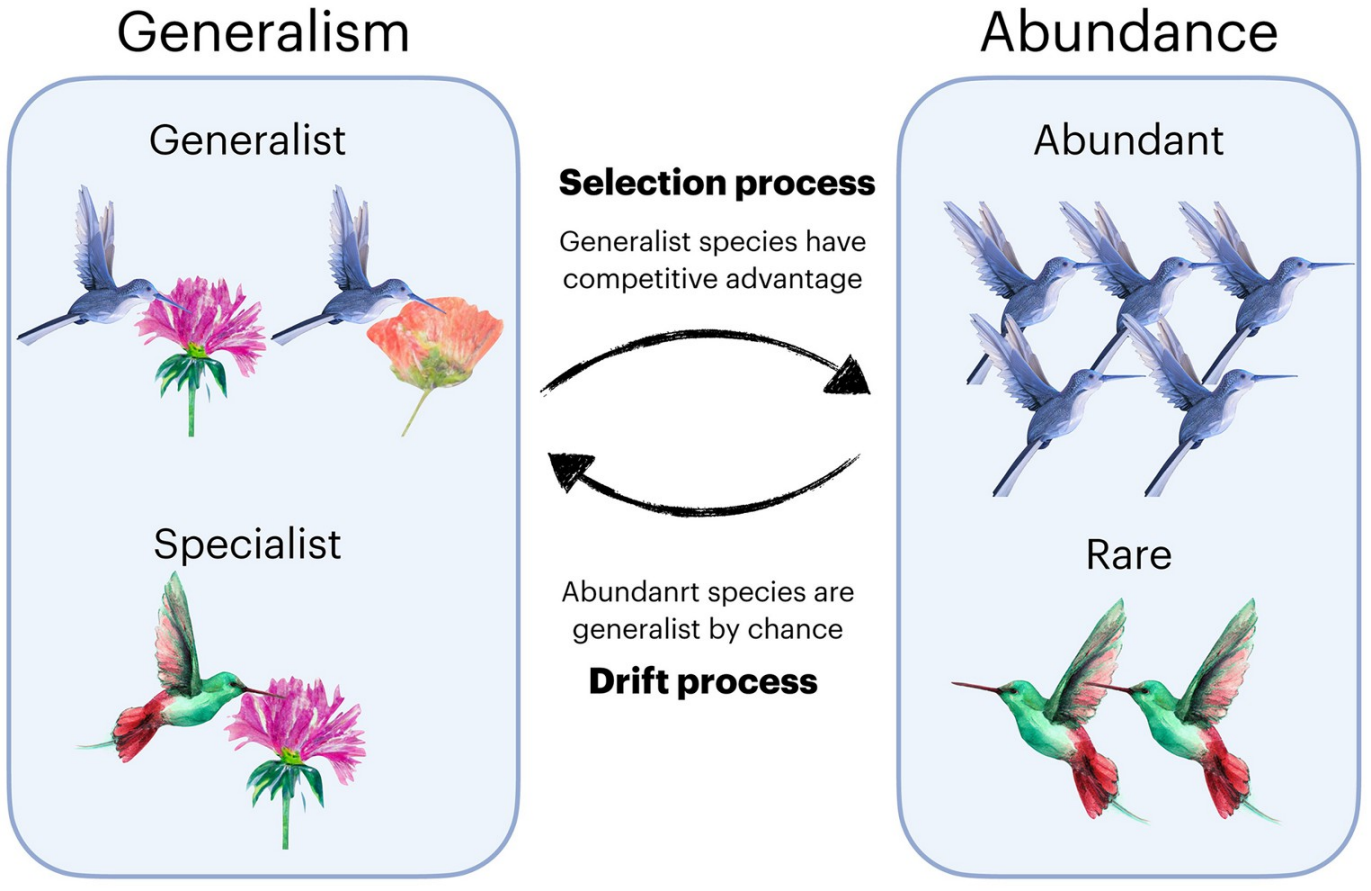
A chicken-and-egg dilemma of generalism and abundance. Empirical evidence shows that abundant species are also generalists. However, the causal direction is debated. If the community is mainly structured by selection processes, then species are more abundant because generalists have a competitive advantage. In contrast, if the community is mainly structured by drift processes, then species are more generalized because abundant populations have a higher chance of encountering more partners. The clip-arts of flowers and hummingbirds are made with DALL·E.

Here, we examine the role of species generalism as a predictor of abundance. Generalism is the (biotic or abiotic) niche breadth of a species [19, 20]; this is an archetypal feature of a species that strongly correlates with its abundance [21]. Specifically, we focus on biotic niche breadth and we will refer to the number of interacting partners in an interaction network as a measure of breadth. Despite the strong correlation, identifying the causal direction between abundance and generalism is not a trivial problem. Indeed, we have a chicken-and-egg dilemma: both causal directions make intuitive sense, and it is difficult to discern *a priori* which direction is correct. Specifically, whether being a generalist causes a species to be more abundant, or whether being more abundant causes a species to be more generalized [3, 21–25]. These two causal directions present fundamentally different views on multispecies dynamics in a local community (Fig 1). In one causal direction—via selection processes such as resource limitation (niche-based)—generalist species are competitively advantaged by having access to a wider range of resources, which causes higher abundance. In the other causal direction—via drift processes (neutrality-based)—abundant species are more likely to occupy more biotic niche space simply by coming into contact with more interaction partners than rare species, resulting in greater generalism. To further add to the complexity, the causal direction between abundance and generalism may not be unidirectional because it is unlikely that only selection or drift processes are occurring [26]. However, we do not know whether and when selection or drift processes *predominantly* structure multispecies dynamics in a local community [3, 26]. Thus, understanding the relative causal direction between abundance and generalism would increase our understanding of the roles of selection and drift in the structuring of ecological communities.

We employ a computational approach—without assuming a mechanistic model of how species abundances are generated—to directly identify the *causal* explanations for species abundance and other ecological patterns, such as species generalism. The computational problem of identifying the causal direction is known as *causal discovery* or structural identification in the field of statistics [27]. Note that causal discovery is different from *causal inference*, where causal discovery aims to find the causal direction while causal inference aims to find the causal strength given the preassigned causal directions. While causal inference has become a popular tool in quantitative ecology [28–31], causal discovery remains rarely used in ecology. This is partly because causal discovery is a notoriously difficult problem and has only taken off in the past decade [32–34]. Without any assumption, it is mathematically impossible to correctly distinguish causes and effects [35, 36].

To address this fundamental constraint, researchers in the field of causal discovery have recently developed a set of computational methods that can operate under minimal assumptions of the causal forms (Chapter 4 of [27]). In particular, two methods have a firm theoretical foundation and are widely applicable. One method is the nonlinear additive noise model based on nonparametric regression [27, 37], and the other method is the geometric-information inference based on information theory [38, 39]. Both methods take advantage of “asymmetry” resulting from the one-way causal direction: the nonlinear additive noise model focuses on the asymmetry of noise, while the geometric-information inference focuses on the asymmetry of information. In parallel, in the field of community ecology, a new method with a different theoretical foundation has been proposed to identify the causal direction between generalism and abundance [24]. A key assumption of Fort et al.’s method is the need to classify continuous data into binary categories (e.g. classifying species abundance data into either abundant or rare). However, the method can be sensitive to how the data are binarized, especially given the log-normal nature of species abundance distributions.

We take a computational causal discovery approach to the chicken-and-egg dilemma between generalism and abundance, and we apply three methods with independent theoretical foundations: (i) our refinement on Fort et al.’s method based on formal logic [24], (ii) the nonlinear additive noise model based on nonparametric regression [27, 37], and (iii) the geometric-information inference based on information theory [38, 39]. Our computational approach not only allows us to detect the causal directions, but also to use the relative strength of the causal directions as a proxy of the relative roles of either selection or drift processes. We evaluate the sensitivity of these three methods to plant–hummingbird data across the Americas [25, 40] and reef fishes data from the Reef Life Survey program [41, 42]. All three methods consistently found strong evidence that generalism drives abundance in these plant-hummingbird communities and reef fish datasets. In addition, we found strong evidence that selection processes act more strongly than drift processes when local temperatures are more variable.

## Methods

### Data

We have used two cross-sectional datasets. One dataset of reef fishes contains measures of species abundance and generalism [42]. Species generalism in this dataset empirically estimates the habitat niche breadth of each fish species. The details of how niche breadth is inferred can be found in [42]. The other dataset has 25 plant-hummingbird systems across the Americas, which contains high-quality network structures and independent measures of plant and hummingbird abundance [43]. To empirically test the causal direction, we need independent estimates of species generalism and abundance (e.g., they cannot be both estimated from visitation data) [44, 45]. The details of this plant-hummingbird dataset can be found in [25]. Species generalism can be measured in different ways from network topology [3]. We have adopted two network metrics of generalism: the degree (number of interacting partners) and the normalized degree (the percentage of interacting partners over all locally available partners). These two metrics exhibit strong correlations with species abundance (S1 Data). Note that the generalism of plants is likely to be underestimated because we do not have the full pollinator-plant networks (e.g., insect pollinators are not sampled).

To understand the mechanisms of the causal directions, we further used the structural and environmental factors in the dataset of plant-hummingbird systems. For the structural factor, we computed four network properties: nestedness, modularity, connectance, and motif frequency. Nestedness is a key community property that has been shown to support biodiversity in mutualistic systems [46, 47]. Specifically, we measure nestedness with the combined nestedness metric (NODF_*c*_), because it can provide an unbiased assessment of the level of nestedness across networks [47–50]. Modularity measures the extent to which a network can be divided into subnetworks that are strongly interacting within but are weakly connected across. Specifically, we measure modularity as Newman’s Q value [51]. Connectance measures the number of realized interactions over the number of all possible interactions. Motif frequency measures the relative occurrences of a motif in the whole network [52]. Specifically, we use all motifs with 2 to 4 species.

For the environmental factors, we gathered the temperature data for networks with location information from the public repository Terraclimate [53]. Fig P in S1 Data shows a strong association between the nestedness factor and the temperature factor, confirming previous findings [47].

### Causal discovery method: Refining Fort et al. (2016)’s algorithm

We briefly describe the method proposed by [24]. Suppose species’ abundance and generalism are both binarized. Specifically, the abundance of a species is categorized as either abundant or rare, while the generalism of a species is categorized as either generalist or specialist. This combines to give us four qualitative categories. Given the strong association between abundance and generalism, we would frequently observe two combinatory categories—species being abundant and generalist concurrently or being rare and specialist concurrently—regardless of the causal direction between abundance and generalism. The crux of this method is that the causal direction would determine the frequency of the other two combinatory categories. If abundance causes generalism, then we are unlikely to observe species being abundant and specialist concurrently. In contrast, if generalism causes abundance, then we are unlikely to observe species being rare and generalist concurrently.

The justification of Fort el al.’s method [24] relies on the formal logic of binarized categories. However, abundance and generalism are continuous gradients. To address this issue, [24] has proposed two methods to binarize continuous data of abundance and generalism, which was further refined in [25]. We follow the refinement proposed by [25]: specifically, a species is classified as abundant with probability of the rescaled abundance $\frac{\text{abundance}-\text{min}(\text{abundance})}{\text{max}(\text{abundance})-\text{min}(\text{abundance})},$ and classified as rare with one minus the rescaled abundance. Similarly, we can classify species into generalist and specialist: a species is classified as generalist with probability of the rescaled generalism $\frac{\text{generalism}-\text{min}(\text{generalism})}{\text{max}(\text{generalism})-\text{min}(\text{generalism})},$ and classified as specialist with one minus the rescaled generalism. However, the argument of the formal logic on the binary variables does not immediately provide a justification of this new methodology on probabilistic variables. Thus, we argue that it needs to be tested with a proper null model [54].

We generate, therefore, a simple null model to show that the original Fort el al.’s method [24] is sensitive to nonlinear causal relationships. We consider two cases where the variable *X* causes variable *Y*. In the first case, *X* is generated from a uniform distribution, while *Y* is generated as *X* plus some noise, where the magnitude of the noise decreases as *X* increases. The decreasing magnitude of the noise fills the assumption in the method (i.e., *X* being large and *Y* being small are unlikely to co-occur), as follows

$$\begin{array}{c} \begin{array}{cc} x & ∼\text{Uniform}(0,1),\\ y & ∼x+\text{Normal}(0,g(x)), \end{array} \end{array}$$ (1)

where *g*(*x*) is a monotonically decreasing function of *x*. In the other case, everything remains the same except that *Y* is generated from *X* with a non-linear function. Formally, we have

$$\begin{array}{c} \begin{array}{cc} x & ∼\text{Uniform}(0,1),\\ y & ∼x^{2}+\text{Normal}\left(0,g\left(x\right)\right). \end{array} \end{array}$$ (2)

The key difference between these two cases lies in the marginal distributions of the effects *Y*. In the first case, the noise induces more “large” values of *Y* than “small” values (Panel A in Fig B in S1 Data). In the second case, the presence of nonlinear causal relationships reverses this pattern, inducing more “small” values of *Y* than “large” values (Fig Panel C in Fig B in S1 Data). As a result, we would expect to see more species with small *x* and large *y* concurrently in the first case (indication of *X* causing *Y*), while more species with large *x* and small *y* in the second case (indication of *Y* causing *X*). However, the ground truth is that *X* causes *Y* in both cases. Despite the appeal of the method when applied to binary data, the original Fort el al.’s method [24] essentially compares which variable has a more skewed marginal distribution when applied to continuous data. In other words, whether species abundance, without reference to its generalism, is more skewed, or vice versa. This is not ideal as this causal discovery method should be based on the joint distribution (i.e., the association between abundance and generalism) instead of the marginal distribution (i.e., the isolated property of either abundance or generalism).

The potential bias of the method can be remedied by rescaling the marginal distributions. We first consider the marginal distribution of species abundance. The lognormal distribution of species abundance is one of the few universal patterns in community ecology [55]. The dataset we use unsurprisingly exhibits lognormal species abundance distribution (Fig E in S1 Data). We then consider the marginal distribution of generalism. Unlike species abundance, the distribution of generalism is not log-normal (Fig F in S1 Data). If we use the degree (number of interacting partners) as a measure of generalism, the distribution of degree commonly follows a truncated power law $P(k)∼k^{-\gamma }e^{k/k_{c}}$, where *k* is the degree, *γ* is the critical exponent, and *k*_*c*_ is the cutoff degree [56–58]. However, the distribution of normalized degree (i.e., the percentage of interacting partners over all locally available partners) does not tend to follow a truncated power law. Thus, we face a problem of model selection requiring us to transform different distributions with different metrics.

**Fig 2:**
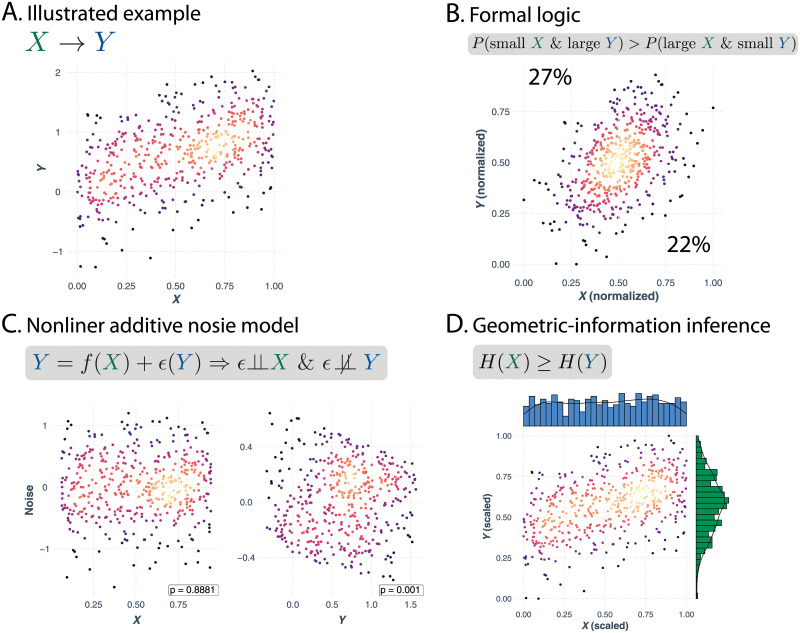
Illustration of the causal discovery methods. Panel (**A**) shows a hypothetical dataset where *X* is the cause and *Y* is the effect. We generate this dataset as an ensemble *y* ∼ *x*^*d*^ with white noise, where *d* ranges from 0.5 to 1. The color of the points represents the density of data: the darker it is, less points it represents. Panel (**B**)-(**D**) illustrate three causal discovery methods we adopted. Panel (**B**) illustrates the method based on formal logic. The criteria of *X* being the cause is that the proportion of points with small *x* and large *y* values is greater than that of points with large *x* and small *y* values. The marginal distributions are both normalized to keep roughly equal proportions of small and large samples. Normalization can help avoid the bias in the method regarding skewed marginal distributions. Panel (**C**) illustrates the method based on nonlinear additive noise model. This method assumes that the effect (i.e., *Y*) is some potentially nonlinear transformation of the cause (i.e., *f*(*X*)) plus some noise that is independent of the cause (i.e., *ϵ*(*Y*)). The criteria of *X* being the cause is that the residuals from the regressions should be independent of *X* but not of *Y*. The form of the nonlinear transformation *f*(*X*) and consequently the residuals can be fitted through nonparametric regressions. Panel (**D**) illustrates the method based on information theory. The criteria of *X* being the cause is that *X* has a higher entropy than *Y*. The marginal distributions are both scaled via a linear transformation between 0 and 1 for a fair comparison of entropy.

To solve this issue, we use a semiparametric approach to transform the skewed marginal distributions of both species’ abundance and generalism [59]. The marginal distribution is transformed into a normal distribution with a mean 0.5. The choice of the mean is to balance the proportions of “small” and “large” values. We then follow the same procedure adopted in the previous method by [25] to normalize the values into probability. Fig 2B illustrates this method. Importantly, the transformation of the marginal distributions preserves the strong association between abundance and generalism in the joint distribution (Fig A in S1 Data). Fig C in S1 Data shows, with simulated data, that our refined method works for some ecologically relevant nonlinear causal relationships.

### Testing other methods of casual discovery

To provide stronger confidence in the detected causal directions, we used two widely adopted methods in causal discovery that are distinct from Fort et al.’s method [24]: nonlinear additive noise model and information-geometric inference [27, 37]. Here, we provide a succinct summary of these methods.

First, we focus on the method based on nonlinear additive noise model. Suppose the variable *Y* is caused by variable *X*. This method assumes that *Y* is generated by a (potentially nonlinear) function *f* of variable *X* with some noise *ϵ* that is independent of *X*. Formally, we can write it as

$$\begin{array}{c} Y=f(X)+\epsilon ,\;\epsilon ⫫X. \end{array}$$ (3)

The assumption of additive noise is common in ecological models. For example, additive noise may represent measurement error [60–62] or stochastic dynamics [63, 64].

This method takes advantage of the asymmetry of noise. Specifically, the noise *ϵ*(*Y*) is only independent of *X* but not independent of *Y* if *Y* is caused by *X*. Vice versa, the noise *ϵ*(*X*) would only be independent of *Y* but not of *X* if *X* is caused by *Y*. Thus, by testing whether the residuals are independent of *X* or *Y*, we can know which variable is the cause. To implement this method, we first fit the nonlinear generating function using the non-parametric generalized additive model [65, 66]. We then calculate the residual and use the kernel-based tests for independence [67, 68]. Details can be found in S1 Data. Fig 2C illustrates this method.

Then, we focus on the method based on information-geometric inference. Suppose again that the variable *Y* is caused by variable *X*. This method assumes a deterministic mapping between *X* and *Y*. More formally, we have *Y* = *f*(*X*, *ϵ*), where *f* is any diffeomorphism (i.e., bejective and its inverse is differentiable) function and the noise *ϵ* is constant (note that noise does not have to be additive as in Eq 3).

This method takes advantage of the asymmetry of information [38, 39]. Intuitively, if *X* causes *Y* in a nonlinear manner with noise, then some information in *X* would be lost in *Y*. More formally, the marginal distribution of *Y* tends to peak when *f* has a small slope (i.e., there is negative correlation between *P*(*Y*) and *f*′), while the marginal distribution of *X* does not correlate with the slope of *f*. Researchers have proved that this result can be equivalently expressed as an entropy-based criterion. Formally, if *X* causes *Y*, we have

$$\begin{array}{c} H(X)\geq H(Y), \end{array}$$ (4)

where *H* denotes the differential Shannon entropy. Vice versa, if *Y* causes *X*. To implement this method, we estimate the empirical entropy of *X* via [69]:

$$\begin{array}{c} \hat{H}\left(X\right)=\psi \left(m\right)-\psi \left(1\right)+\frac{1}{m-1}\sum_{i=1}^{m-1}\;log\left(x_{i+1}-x_{i}\right), \end{array}$$ (5)

where $\psi =\frac{\text{d}\;\text{ln}\Gamma (X)}{\text{d}x}$ is the digamma function, *x* are ordered ascendingly (*x*_*i*_ ≤ *x*_*i*+1_) and rescaled to be bounded between 0 and 1, and log(0) is defined as 0. The empirical entropy $\hat{H}(Y)$ can be similarly calculated. Details can be found in S1 Data. Fig 2D illustrates this method.

## Results

**Fig 3:**
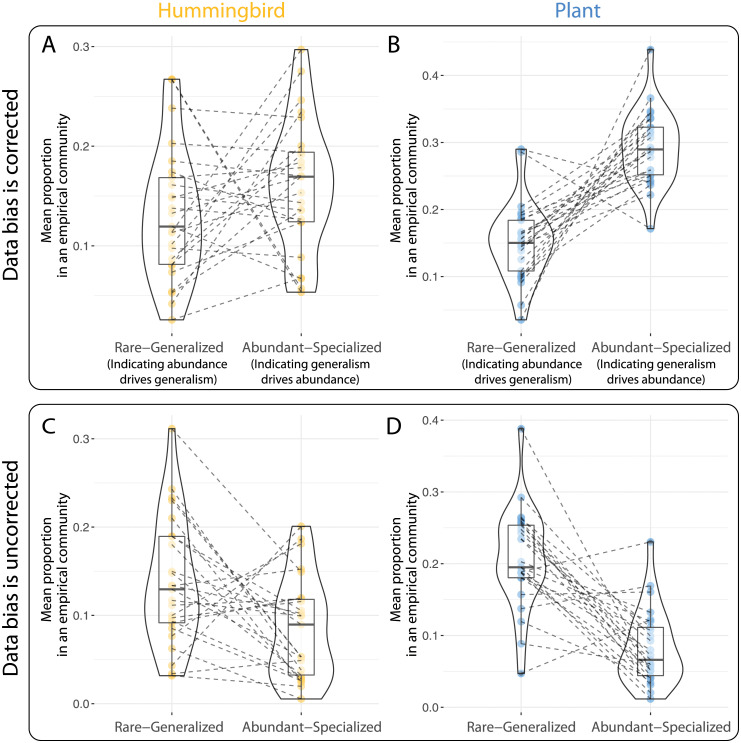
Generalism drives abundance. We apply the causal discovery method based on formal logic to detect the causal direction in an empirical dataset of 25 hummingbird-plant communities. In each panel, the *x* axis shows the two categories (rare and generalized species versus abundant and species species), and the *y* axis shows the mean proportion of species in that category. Each point denotes a different empirical community. Each line connects two points in the same empirical community. If the line is going up (meaning there are more species being abundant and specialized), it indicates that generalism drives abundance, and vice versa. The original method (panels **C** and **D**) suggests that generalism drives abundance in most communities (this is expected because the marginal distribution of abundance is more skewed than that of generalism). In contrast, our refined method has removed the bias regarding skewed marginal distributions, and it (panels **A** and **B**) suggests that generalism drives abundance in most communities. The qualitative patterns are similar among hummingbirds (panel **A**) and plants (panel **B**).

Using our refinement of Fort et al.’s method [24], we found that generalism drives abundance for species in both datasets. For the sake of brevity, we focus on the results of the plant-hummingbird system here and refer the reader to S1 Data for the details of the results obtained with the reef fishes dataset. For the dataset of plant-hummingbird systems, Fig 3A and 3B show that the proportion of species that are abundant and specialist concurrently (indication of generalism as the cause) is generally higher than the species that are rare and generalist concurrently (indication of abundance as the cause). Specifically, 19 out of 25 communities exhibit evidence that generalism is the cause in these hummingbird species, while 23 out of 25 communities exhibit such evidence for plant species. This result is not obtained when analyzed with the original Fort el al.’s method [24] (Fig 3C and 3D). This contrast is expected given that the original method is biased to assign the variable with a more skewed marginal distribution as the cause (see Fig G in S1 Data with the transformed unskewed marginal distributions). Formal analysis of statistical significance can be found in S1 Data.

**Fig 4:**
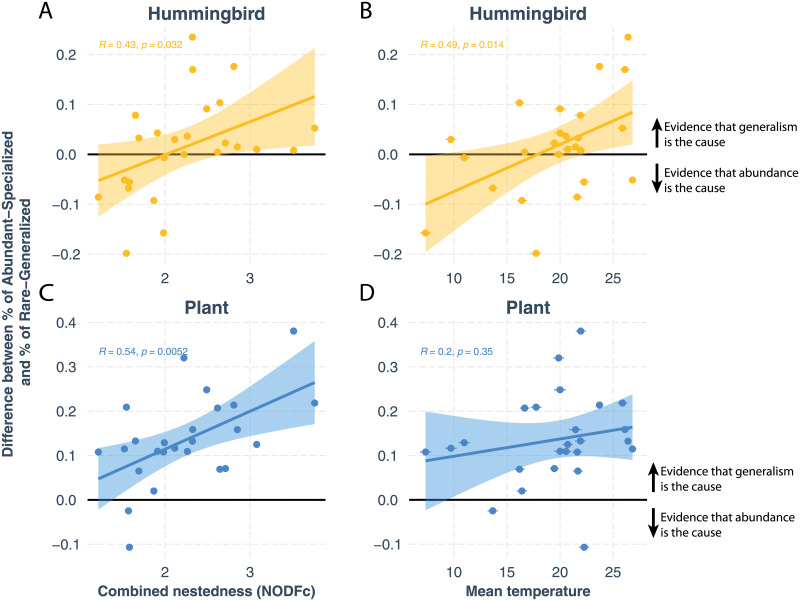
Strength of selection process versus drift process under structural and environmental context. Panels (**A**) and (**C**) show the effects of the structural context. The *x* axis shows the combined nestedness, which is an unbiased metric to compare the level of nestedness across networks [47, 50]. The *y* axis shows the differences between the proportion of abundant and specialist species and that of rare and generalist species. A positive difference indicates that generalism is the cause of abundance (i.e., stronger selection and weaker drift), while a negative difference indicates that abundance is the cause of generalism (i.e., weaker selection and stronger drift). Each point denotes a different empirical plant-humming bird community (i.e., dataset). The orange points represent the hummingbird data while the blue points represent the plant data. The strength of the selection processes increases as the communities have more nested structures for both plants and hummingbirds. Plants exhibit stronger positive patterns than hummingbirds under the structural context. Panels (**B**) and (**D**) show the effects of the environmental context. The *x* axis shows the local annual temperature mean where the community was sampled. The error bar represents 1 standard deviation with temperature mean from 1958 to 2020 [53]. The strength of the selection processes increases as the communities experience higher mean temperature for both plants and hummingbirds. Hummingbirds exhibit stronger positive patterns than plants under the environmental context. The Pearson correlations and their *p* values are shown in the figure.

In addition, these exceptions, where abundances appear to be the cause, only occur when the communities are structured with poorly nested networks and under relatively low environmental variation. Fig 4(A) and 4(C) shows a strong correlation between the level of nestedness and the relative strength of the causal direction [48, 50]. Specifically, the causal evidence for generalism as a driver of observed patterns, especially for plants, is stronger when the community has a more nested structure. Among four structural factors we studied (nestedness, modularity, connectance, and motif analysis), nestedness shows the most strong association with the causal strength (see S1 Data for details). Fig 4(B) and 4(D) shows a strong correlation between the mean temperature and the relative strength of the causal direction. Specifically, the causal evidence for generalism as a driver of observed patterns, especially for hummingbirds, is stronger when the community is under greater environmental variations. Further statistical analysis with dimension reduction and nonparametric statistics can be found in S1 Data.

The casual discovery approaches based on additive noise model and information-geometric inference support the result that generalism drives abundance. With the additive noise model, we found that generalism is likely to be the cause (*p* value = 0.73 for animals and = 0.64 for plants) while abundance is highly unlikely to be the cause (*p* value <10^−3^ for both animal and plants). More details on the model fit (including uncertainty estimation) can be found in S1 Data. With the information-geometric inference, we found that the marginal distributions of generalism are much higher than those of abundance, which indicates that generalism is more likely to be the cause (S1 Data). The results for the reef fishes dataset are qualitatively the same for all methods (S1 Data).

## Discussion

We have studied whether abundance drives generalism or the other way around via a computational causal discovery approach. We have used three independent methods of causal discovery: a refined method of Fort et al.’s based on formal logic [24], the nonlinear additive noise model, and the geometric-information inference. We have found strong evidence that generalism causes species abundance in both datasets of plant-hummingbird communities and reef fishes. We have also found that the causal evidence for generalism as a driver of observed patterns is stronger when the community is exposed to greater environmental variation.

Our results shed light on the big question of selection versus drift processes in structuring *multispecies* dynamics [26]. Since Hubbell’s groundbreaking work [55], this question has taken a central place in community ecology. In *two-species* communities, a fruitful research line has increased our understanding of this problem by rigorously linking experiments and theory [70–72]. Yet, we lack a full understanding of this question in multispecies communities, because it is challenging to carry out experiments that control species interactions in large communities [3]. Our computational causal discovery approach provides an alternative, practical path to tackle this problem in multispecies communities. This approach takes advantage of the fact that causal directions are different between abundance and generalism when the community is structured by selection or drift processes. Thus, based on our causal discovery methods, we found strong evidence that selection processes have a critical role in maintaining species persistence in mutualistic systems. Importantly, while previous works have addressed this question [24, 25], we reverse the previously established conclusion that abundance is the cause by fixing a methodological issue.

Causal discovery is not a novel topic, but a computational approach to causal discovery has only taken off in the last decade [27]. Without doubt, the gold standard in casual discovery is controlled experiments [73, 74]. However, controlled experiments can be difficult or even impossible to conduct in many contexts. These contexts create an opportunity for computational tools to detect the correct causal directions. In ecology, the most adopted tool of causal discovery is convergent cross mapping [75]. While this method has been widely applied to many ecological questions [76–80], this method only works for time series data. The field of causal discovery has recently developed a line of methods that work with cross-sectional data, such as the additive noise model and the method of information-geometric inference. These methods have already been applied to many disciplines, including genetics [81], earth system science [82], and kinetic systems [83]. Yet, to the best of our knowledge, these methods have not been used in ecology. We have demonstrated that these methods are useful in ecological contexts. Importantly, these methods are flexible and can be easily adapted to different datasets. As a proof of its flexibility, we have applied the same methods to analyze the dataset of plant-hummingbird communities and the dataset of reef fishes.

We acknowledge that the causal direction between abundance and generalism is not unidirectional and feedback may occur [3]. As the causal direction represents either selection or drift processes, it is unlikely that only one process is in play. As with many debates in ecology, the reality might lie somewhere along the spectrum of the dichotomy. As the strengths of the causal directions indicate the relative roles of the selection and drift process, our results suggest that selection processes are stronger when the network structure of the community has a higher level of nestedness. Our results are consistent with the literature on this topic. At the species level, empirical evidence in reef fishes [42, 84] and corals [85] shows generalism is favored under variable environments. At the community level, empirical evidence on plant-pollinator communities shows that networks are more nested when located in more variable environments [47, 86, 87].

A limitation of our work is that we did not consider potential confounding factors. Our approach can be heuristically justified by the central limit theorem and our removal of the skewness in marginal distributions (i.e., the ordered quantile normalization [59] in the refined method of Fort et al. [24] or post-linear regression [66] in the additive noise model). To explain the emergence of skewed distributions [13], a simple but general argument is based on the central limit theorem: as the mean of the *summation* of many independent or weakly dependent processes result in a normal distribution, the mean of the *product* of these processes results in a log-normal distribution. In this sense, removing the skewness of the distribution transforms the statistical nature of the distribution from the product of multiple processes into the summation of multiple processes (on an appropriate scale). Thus, by removing the skewness of the marginal distribution via the log transformation or the more general semiparametric transformation [59], we can identify the causal direction with more confidence. Of course, this explanation is far from being rigorous, as we may still suffer from the bias of unobserved confounding factors [88], the omitted-variable bias [89], and that the central limit theorem requires asymptotic aggregations (although there is empirical evidence that small ecological systems can exhibit asymptotic behaviors [90]). A more satisfactory solution is to adopt rigorous methods capable of detecting nonlinear causal directions with the presence of hidden confounding factors. For example, using instrumental variables [88] or the DeCAMFounder method [91]. These methods generally require a sufficiently large amount of data to ensure statistical convergence, which is unfortunately beyond the reach of the datasets we used. Future research with larger datasets should explore these methods to control for hidden confounding factors.

From a broader perspective, we showcase the power of the computational causal discovery approach in ecological research. In the realm where experiments and theory are difficult to apply to estimate the direction of causality, these computational methods show great promise. Meanwhile, the computational identification of causal association can help refine theoretical assumptions and experimental designs for multispecies communities. The association between abundance and generalism we studied here is by no means an exceptional pattern in community ecology. For example, species abundances are also strongly associated with geographic distributions [92]. The flexibility of these computational methods may be similarly applied to study these patterns in the context where experiments have already been conducted [93]. We hope our work can raise the awareness of this causal discovery approach in the era where much ecological data is becoming available [94, 95].

## Supporting information

S1 Data Detailed methods and additional validations, and supplementary figures and tables. (PDF)
