# Supplementary material for: Generalism drives abundance: A computational causal discovery approach
Source: PLoS Comput Biol. 2022 Sep 29;18(9):e1010302. doi: 10.1371/journal.pcbi.1010302 (PMC9521805; doi:10.1371/journal.pcbi.1010302)
Supplement: S1 Data — (PDF) [file pcbi.1010302.s001.pdf]

Supporting Information for

Generalism drives abundance: a computational causal discovery approach

Chuliang Song[1,2], Benno I. Simmons[3], Marie-Josée Fortin[2], Andrew Gonzalez[1]

[1]Department of Biology, McGill University,
1205 Dr. Penfield Avenue, Montreal, H3A 1B1 Canada
[2] Department of Ecology and Evolutionary Biology, University of Toronto,
25 Willcocks Street, Toronto, Ontario M5S 3B2 Canada
[3]Centre for Ecology and Conservation, College of Life and Environmental Sciences,
University of Exeter, Cornwall Campus, Penryn, TR10 9FE, UK

Contents

S1 Association between species abundance and generalism **S1**

S2 Null model analysis of Fort et al.'s method **S2**

S3 Data distribution **S4**

S4 Analysis with the refined Fort et al.'s method **S7**

S5 Analysis of causal associations with additional network properties **S8**

S6 Analysis of causal associations with temperature **S12**

S7 Analysis with additive noise model and information-geometric inference **S15**

S7.1 Additive noise model S15

S7.2 Information-geometric inference S16

S8 Analysis of the dataset of reef fishes **S17**

S1 Association between species abundance and generalism

We used the degree (number of interacting partners) and the normalized degree (the percentage of interacting partners over all locally available partners) to measure generalism. For both metrics, we found a strong correlation between generalism and abundance in animal species, and a weak but significant correlation in plant species. These correlations only change subtly after we normalize the distributions (Figure A in S1 Text).

Another widely adopted metric is known as the species-level generalisation index g, which is based on the degree of specialization d' [5]. Specifically, following Fort *et al.* [8], the species-level generalisation index g is defined as $1 - d'/d'_{\text{max}}$, where d' quantifies the derivation from a random sampling of all available able interaction partners, and d'_{max} is the maximum possible value of d' given the constraints. We used the R package 'bipartite' to compute the generalisation index g [6]. We found that the generalisation index g has a null correlation with species abundance in both animal and plant species (Figure A in S1 Text).

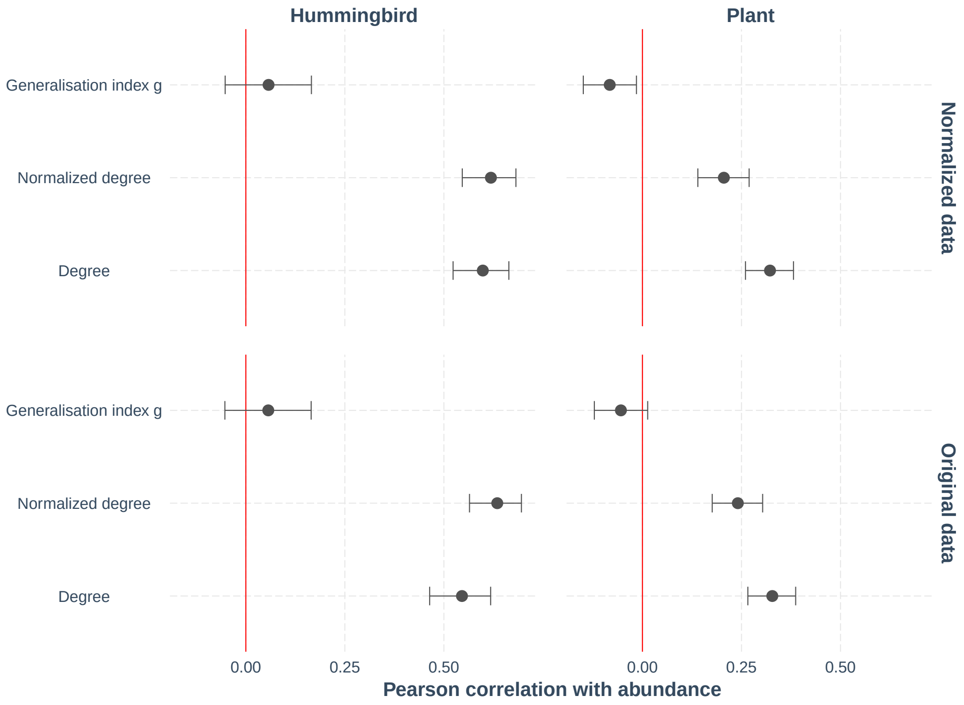

Fig. A. Pearson correlation between abundance and different measures of species generalism. The left panels show animal species while right panels show plant species. The lower panels show the original data while while the right panel shows the transformed unskewed data. The y axis shows the three measures of species generalism. The x axis shows the Pearson correlation between abundance and generalism. The points denote the mean estimated Pearson correlation and the error bar shows the 95% confidence interval.

S2 Null model analysis of Fort et al.'s method

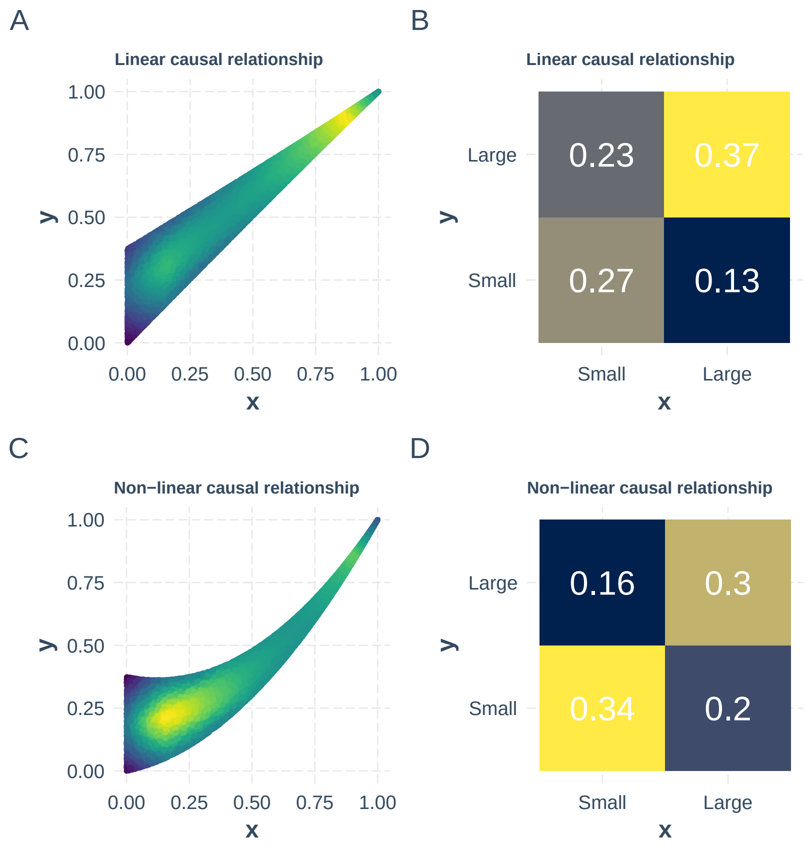

Fig. B. The original method in Fort *et al.* [8] is biased by uneven distributions. Panel (A): y is caused by x with a linear relationship plus some noise. x follows a uniform distribution ranging from 0 to 1. The specific formalism is in Eqn. (1). The color denotes the point density: the more yellow, the point cloud has more points. Panel (C): y is caused by x with a nonlinear relationship plus some noise. x again follows a uniform distribution ranging from 0 to 1. The specific formalism is in Eqn. (2). The original method in Fort *et al.* [8] compares the proportion of points with large x and small y (indication of y being the cause) with the proportion of points with small x and large y (indication of x being the cause). Panel (B): Applying this method to the data in Panel (A), it correctly identified that x is the cause. Panel (D): Applying this method to the data in Panel (C), it incorrectly identified that y is the cause. The reason behind the phenomena is that the non-linear causal relationship skews the distribution of y generating more small y values, which increases the proportion of points with large x and small y.

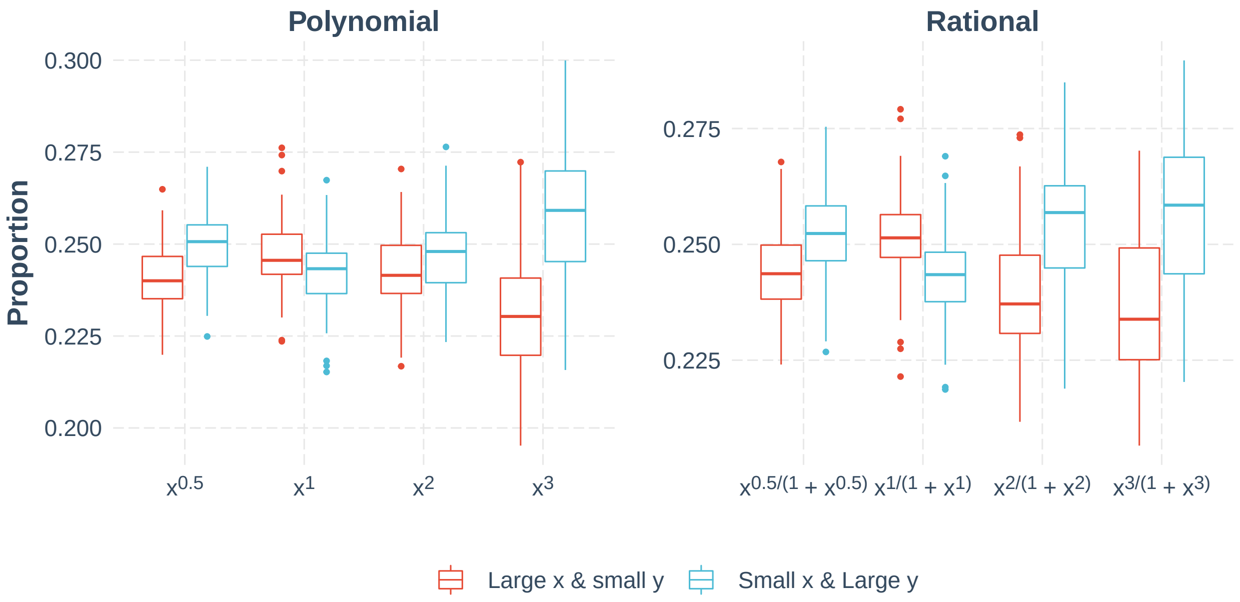

Fig. C. Null model analysis of the refined method of Fort *et al.* (2016). In all analysis, we have that X causes Y. Specially, $Y = f(X) + N(0,1)$, where $N(0,1)$ represents the standard normal distribution. The x axis shows the functional form of the nonlinear causal relationships. The left panel shows the polynomial functional forms, while the right panel shows the rational functional forms. These functional forms represent a range of possible functional responses in ecological literature [2]. The y axis shows the proportion of the two categories in simulated ensembles. The refined method identified the correct cause and effect in most cases.

S3 Data distribution

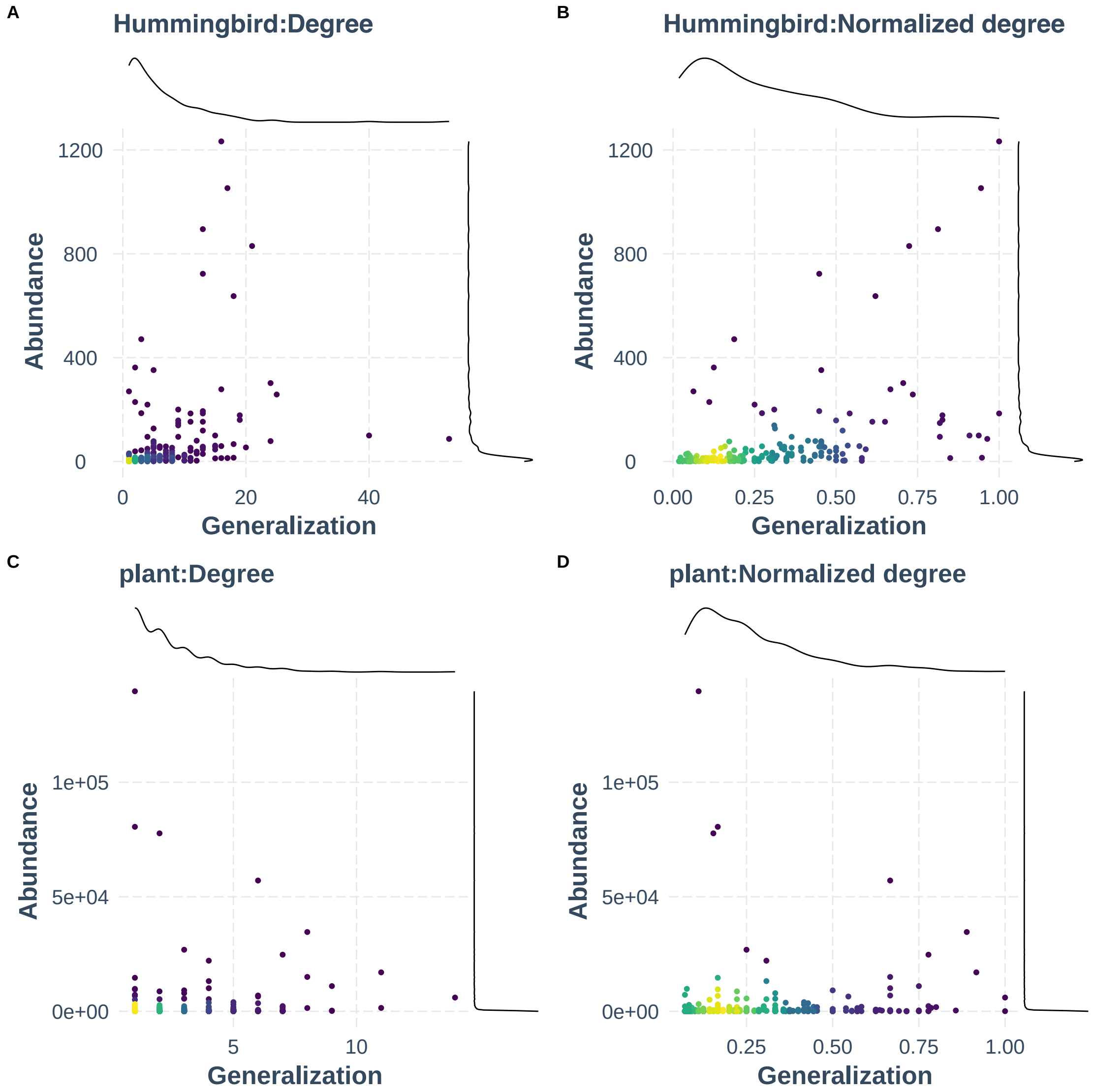

Fig. D. Distribution of original data. The x axis shows the species generalism while the y axis shows the species abundance. Panel (A) shows hummingbird species with the degree as the measure of generalism. Panel (B) shows hummingbird species with the normalized degree as the measure of generalism. Panel (C) shows plant species with the degree as the measure of generalism. Panel (D) shows plant species with the normalized degree as the measure of generalism.

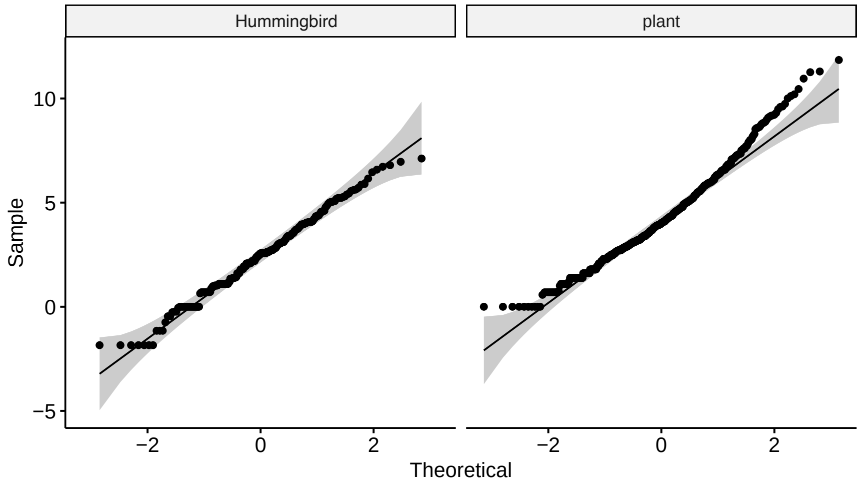

Fig. E. Q-Q plots of abundance in log scale. Q-Q plots show that the abundances of both hummingbird species and plant species largely follow log-normal distributions

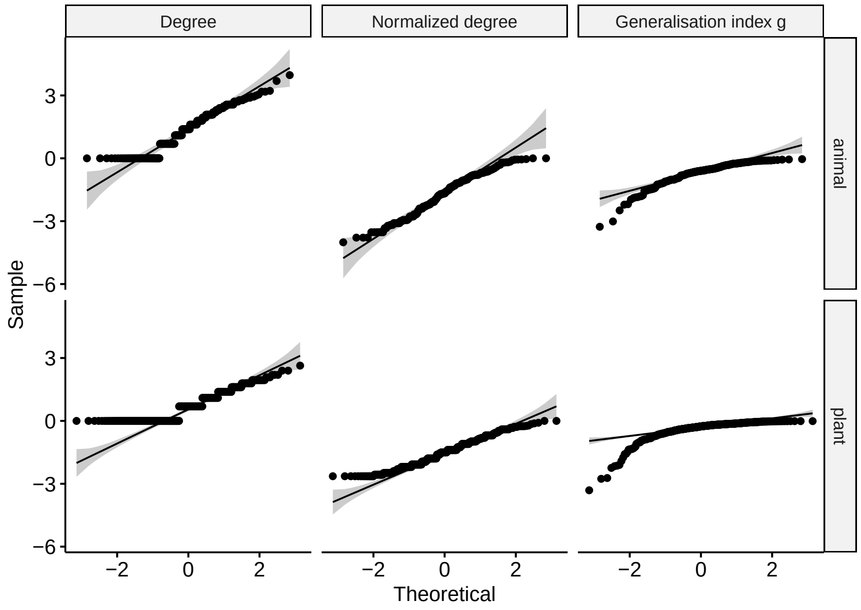

Fig. F. Q-Q plots of generalism in log scale.

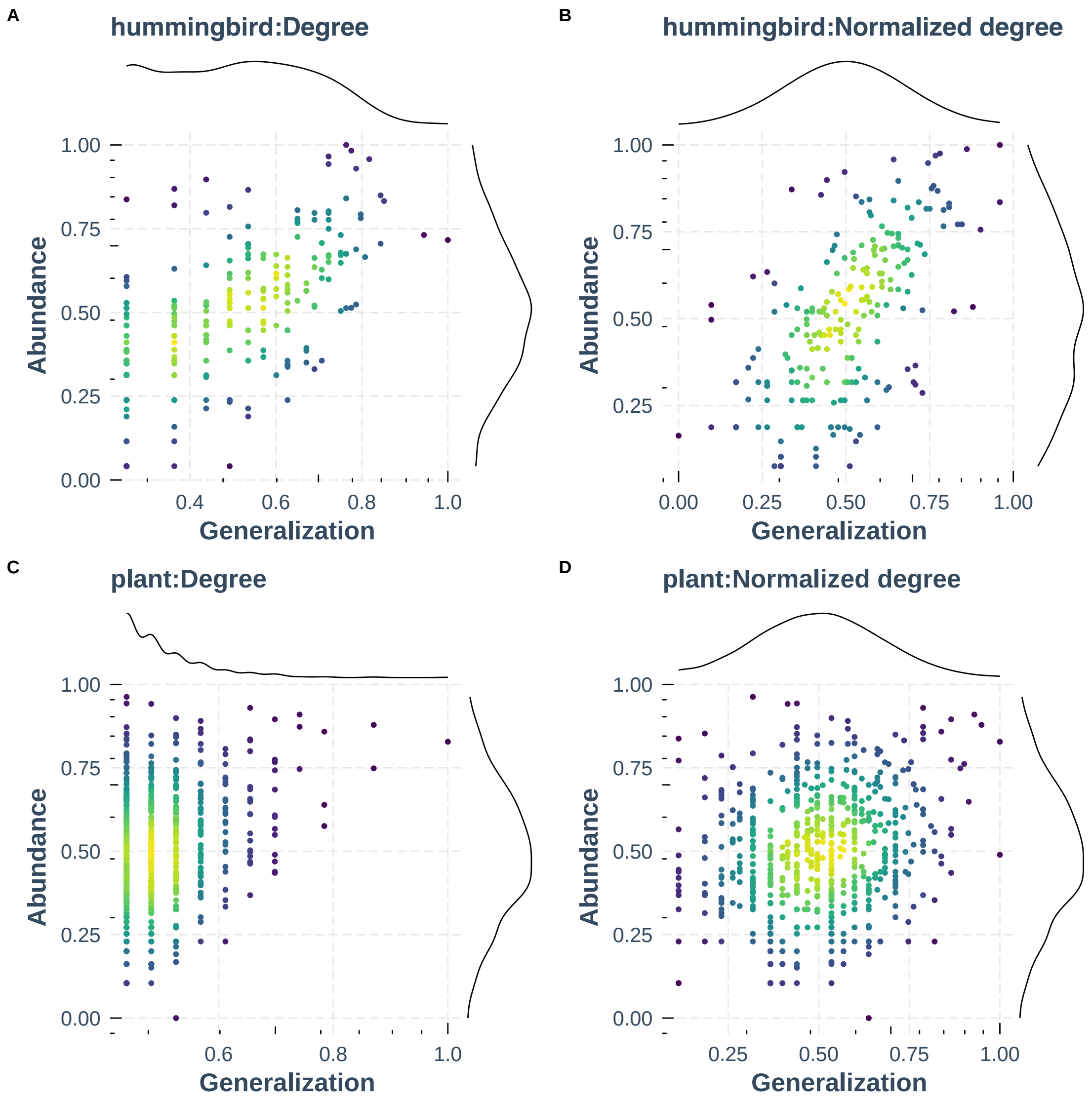

Fig. G. Transformed data with minimal skewness. The original data has high skewness, which could bias the causal discovery methods. We transform the distribution (keeping the ranks of the data fixed) to a normal one with minimal skewness [10].

S4 Analysis with the refined Fort et al.'s method

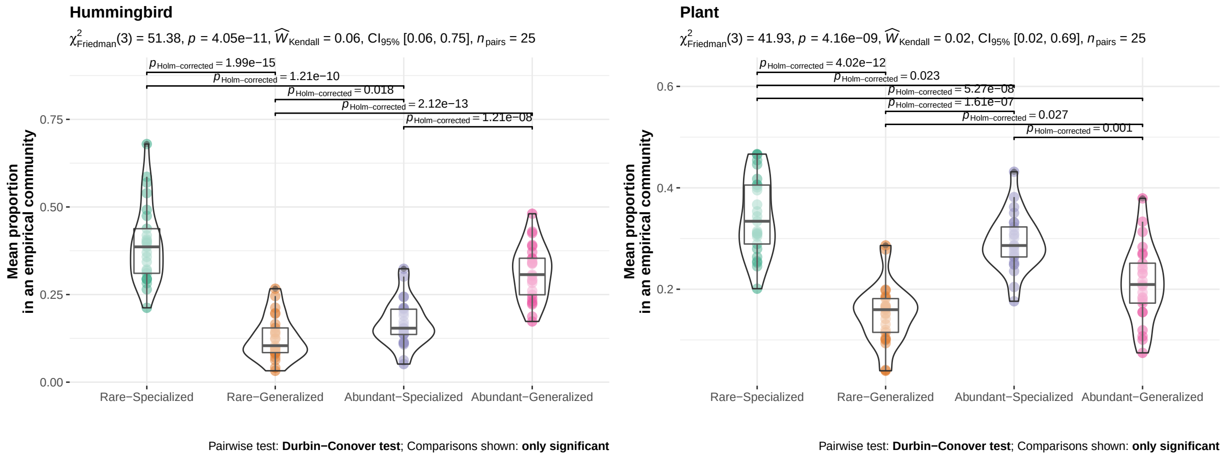

Fig. H. The x axis shows four species types: being rare and specialist concurrently, being rare and generalist concurrently, being abundant and specialist concurrently, and being abundant and generalist concurrently. The y axis shows the mean proportion of each species type. Each point denotes a different empirical plant-hummingbird community. The left axis shows the hummingbird species while the right axis shows the plant species. As expected, the proportions of rare-specialist species and those of abundant-generalist species are high. The proportions of abundant-specialist species are generally higher than those of rare-generalist species (p values are shows in the figure).

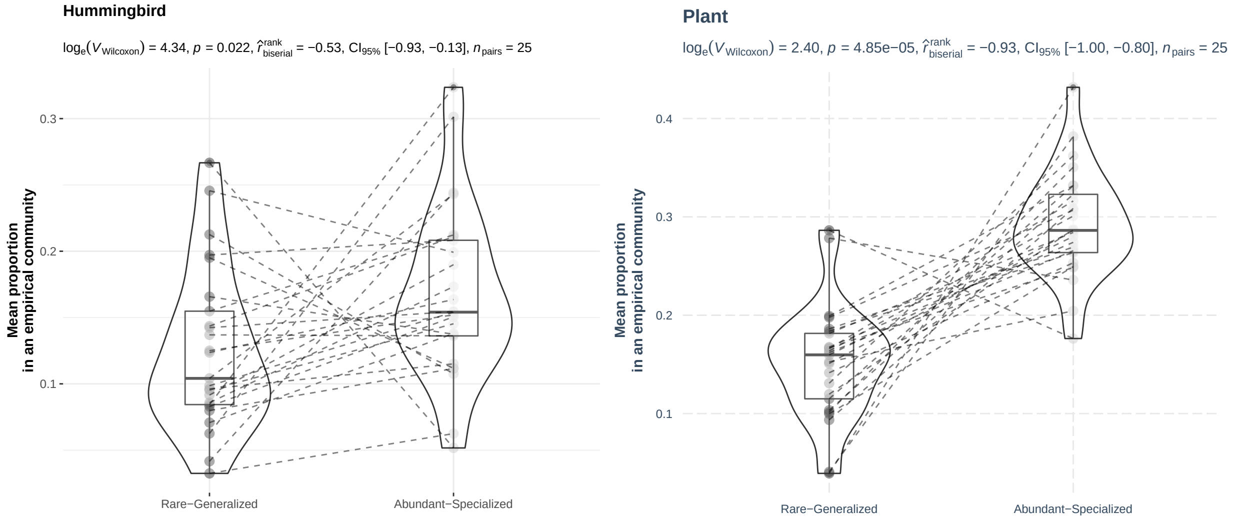

Fig. I. Figure H shows the statistical analysis without considering the proportions are paired. Here, we analyze the proportions of abundant-specialist species and those of rare-generalist species as paired samples. We found again a significant difference that indicates generalism as the cause.

S5 Analysis of causal associations with additional network properties

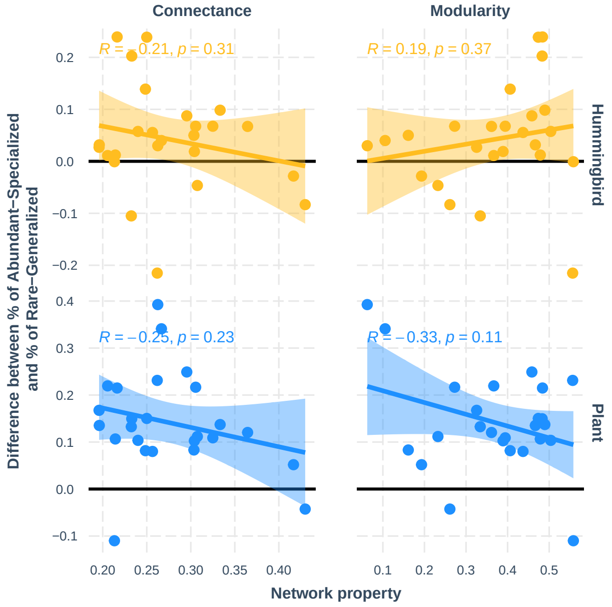

Fig. J. Same as Figure 4 except we replace nestedness with two other network properties: connectance (left column) and modularity (right column). The connectance is defined as the number of realized interactions over the number of all possible interactions. The modularity is defined as Newman's modularity [9], and its value is computed from R package `bipartite` [7]. The corresponding Pearson correlation and its p value are shown in the figure. None of the correlations is statistically significant.

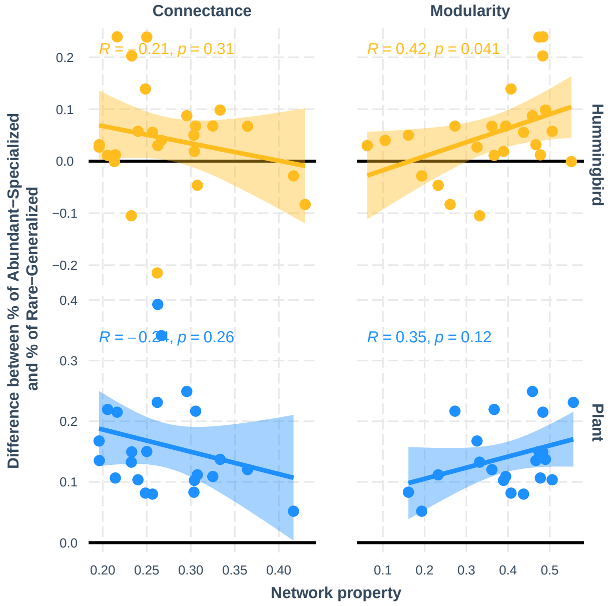

Fig. K. Same as Figure J except we remove the outliers. The outliers are removed if its Cook's distance is larger than 4/# of points. The key qualitative difference from Figure J is that Modularity is now statistically significantly associated with the evidence strength on causal direction.

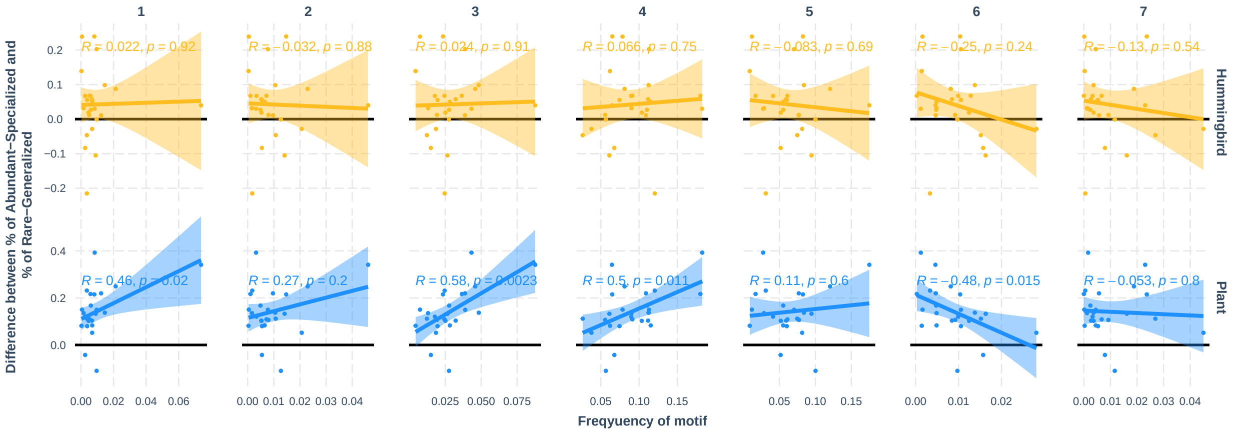

Fig. L. Same as Figure 4 except we replace nestedness with motif frequencies. The horizontal axis shows the frequency of motifs in the bipartite networks, which is computed from the R package `bmotif` [14]. Each column corresponds to a motif, and it is labelled according to Simmons *et al.* 13. The corresponding Pearson correlation and its p value are shown in the figure.

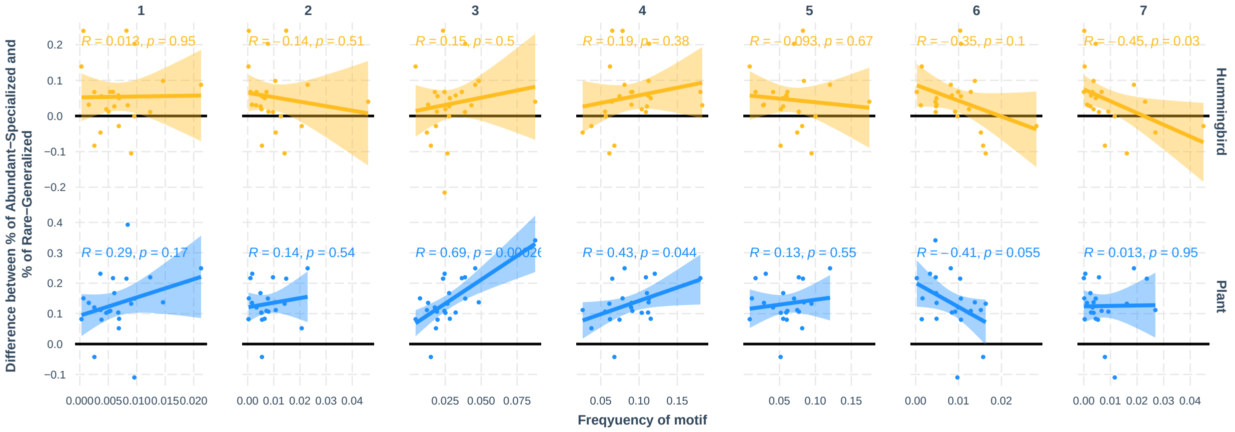

Fig. M. Same as Figure L except we remove the outliers. The outliers are removed if its Cook's distance is larger than 4/# of points.

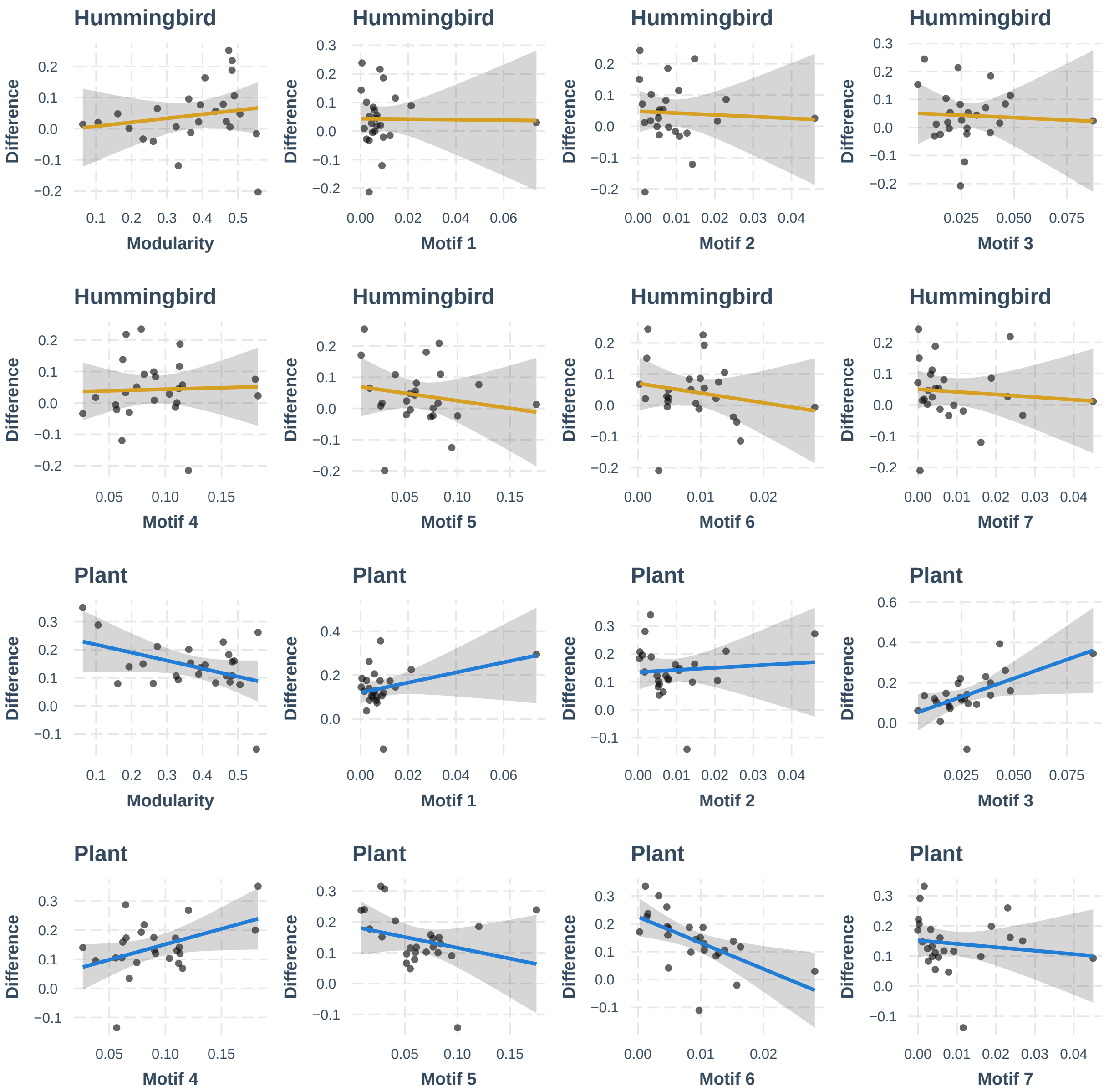

Fig. N. Same as Figures J and L except we control for the effects of connectance and network size. Specifically, we perform linear regression $\mathsf{lm(diff \sim network\ property + connectance + network\ size)}$, where network property represents either modularity or motif frequencies. The partial residuals are plotted. Overall, the qualitative results do not differ from previous analysis.

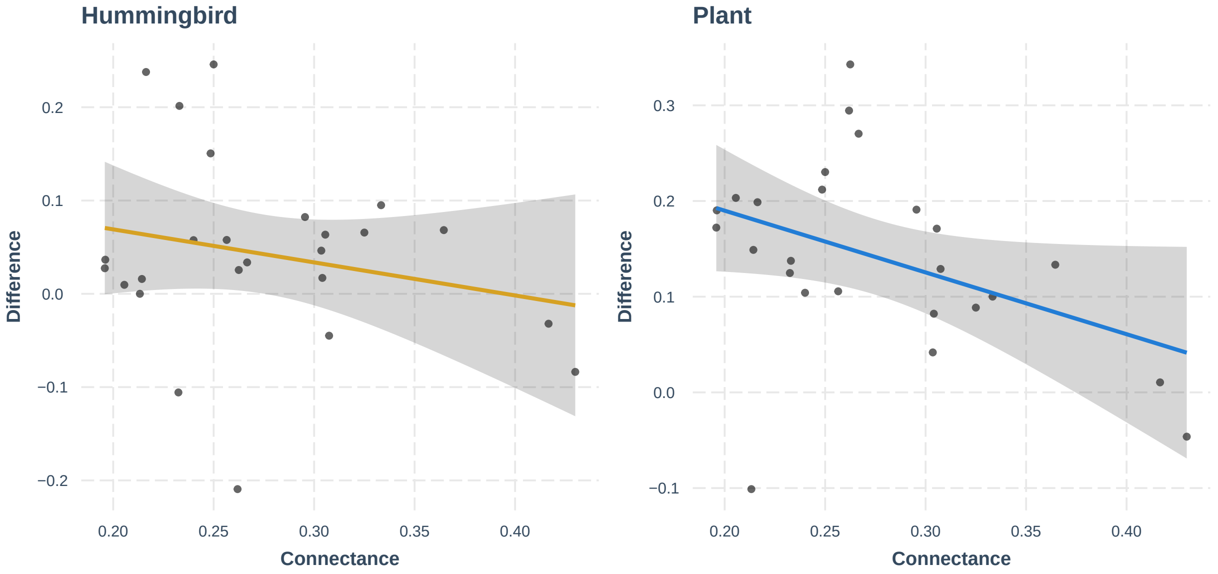

Fig. O. Same as the first column of Figure J except we control for the effects of network size. Specifically, we perform linear regression $\mathsf{lm(diff \sim connectance + network\ size)}$. The partial residuals are plotted. Overall, the qualitative results do not differ from previous analysis.

S6 Analysis of causal associations with temperature

We have gathered the environmental conditions for a subset of networks ($n = 14$) from the public repository `Terraclimate` [1]. Following the methods in Song *et al.* [15, 16], we confirmed a strong association between combined nestedness and local temperature (Figure P in S1 Text).

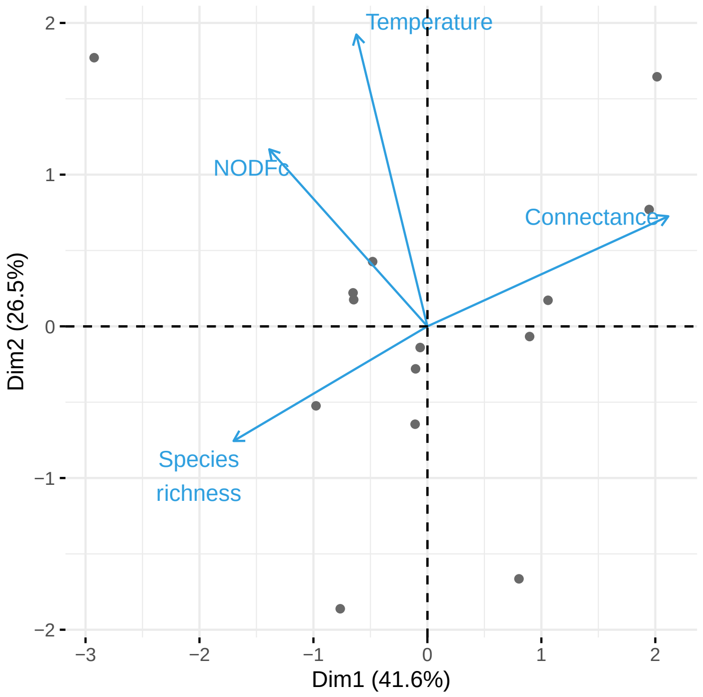

Fig. P. Using a Principal Component Analysis (PCA), the figure shows the two principal components for the four variables investigated: species richness (the geometric mean of plants and hummingbirds), connectance (the proportion of realized interactions among all possible interactions), nestedness statistic and temperature. The arrows correspond to the four associated eigenvectors, and each small dot corresponds to one of the 25 observed networks.

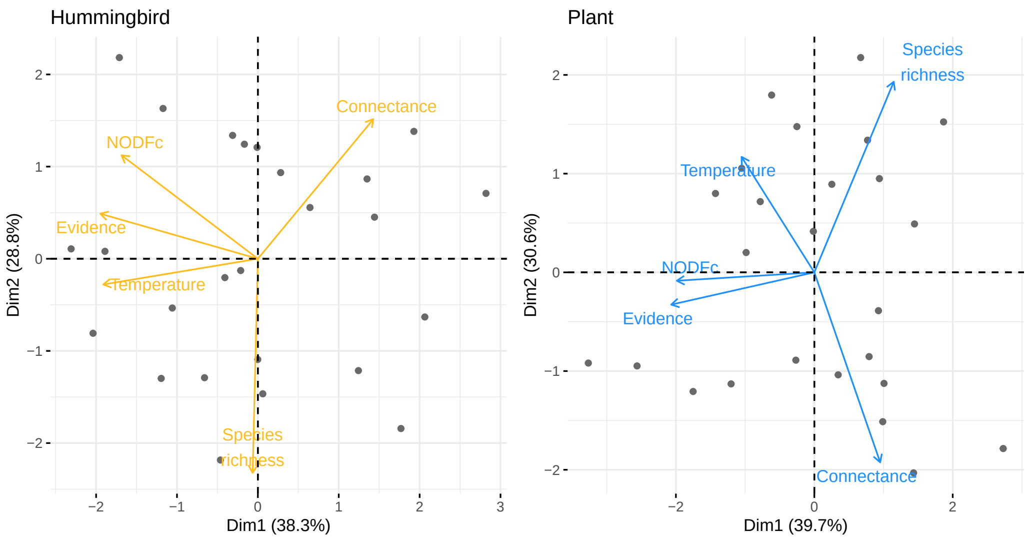

Fig. Q. Using a Principal Component Analysis (PCA), the figure shows the two principal components for the four variables investigated: species richness (the geometric mean of plants and hummingbirds), connectance (the proportion of realized interactions among all possible interactions), nestedness statistic and temperature, and the evidence strength of generalism being the cause. The arrows correspond to the five associated eigenvectors, and each small dot corresponds to one of the 25 observed networks.

We then used a model-free, nonparametric measure of variable association [3]. We used the R package `FOCI` [4]. Figure R in S1 Text confirms the findings in Figure 4.

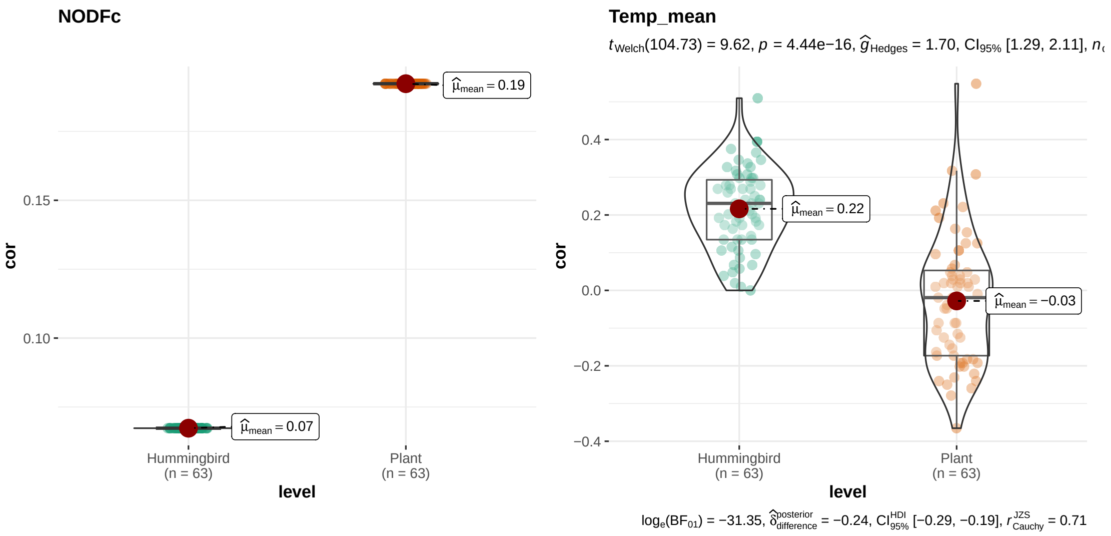

Fig. R. Association between causal direction with structural and environmental context. The left panel shows the structural context (measured as the level of nestedness). The x axis shows the two species categories, hummingbird and plant. The y axis shows the association between the level of nestedness and the causal directions that generalism being the cause [3]. The causal directions in both hummingbirds and plants are both positively correlated with the level of nestedness, albeit plants have a stronger correlation. The right panel shows the environmental context (measured as the mean temperature). The y axis shows The y axis shows the association between the mean temperature and the causal directions that generalism being the cause [3]. The points denote the mean temperature from year 1958-2020 [1]. The causal direction in hummingbirds are positively correlated with the mean temperature, while the direction in plants has null correlation. These results confirm the finding in Figure 4.

S7 Analysis with additive noise model and information-geometric inference

S7.1 Additive noise model

We used a generalized additive models with integrated smoothness estimation [18] from the R package `mgcv` [19]. We used a Gaussian family. In total, we have 228 data points for hummingbirds and 594 data points for plants. We used bootstraps 100 times to estimate the uncertainty.

Figure S in S1 Text shows the adjusted R^2 in the generalized additive models. The models generally have a higher R^2 when generalism is the cause compared to that when abundance is the cause.

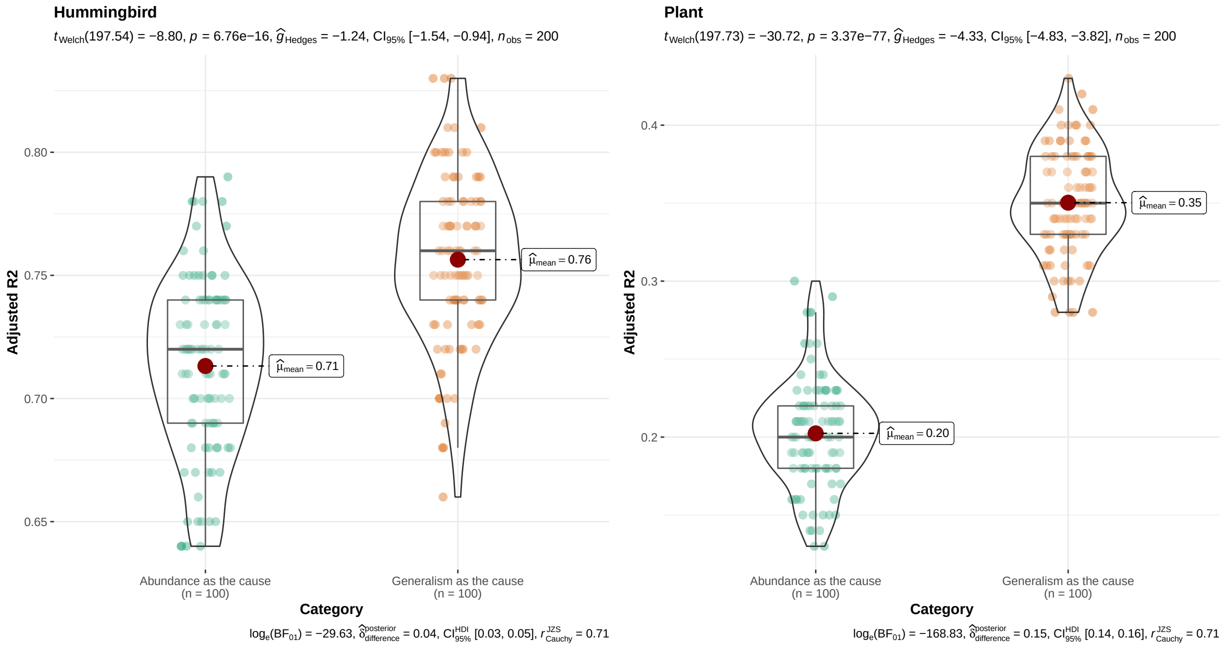

Fig. S. Adjusted R^2 in additive noise models. The x axis shows the two categories of models: one with abundance as the cause (i.e., predictor in the regression), while the other with generalism as the cause. The y axis shows the adjusted R^2 in the system. The points are from 100 boostraps.

We then used the Hilbert Schmidt Independence Criterion to test the independence of the residuals with the variables [11]. We used the R package `dHSIC` [12]. We used the default method with the Gaussian kernel and gamma approximation based test.

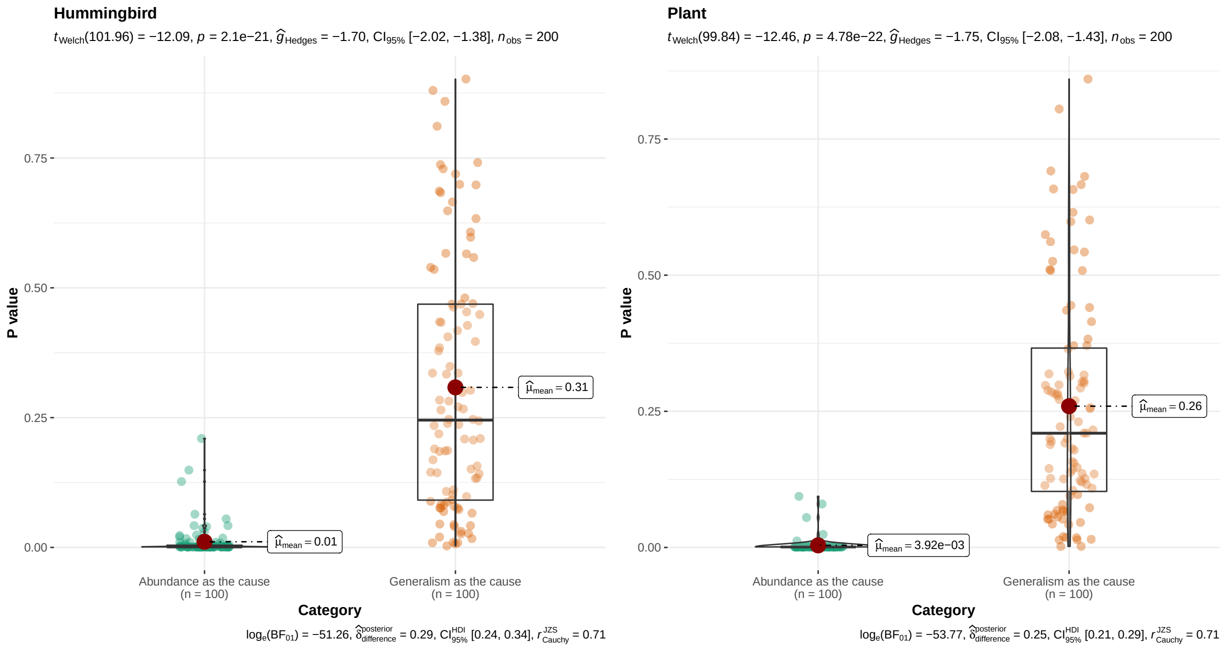

Fig. T. P values in additive noise models. The x axis shows the two categories of models: one with abundance as the cause (i.e., predictor in the regression), while the other with generalism as the cause. The y axis shows p values between the predictor with the residuals in the system. The points are from 100 boostraps.

S7.2 Information-geometric inference

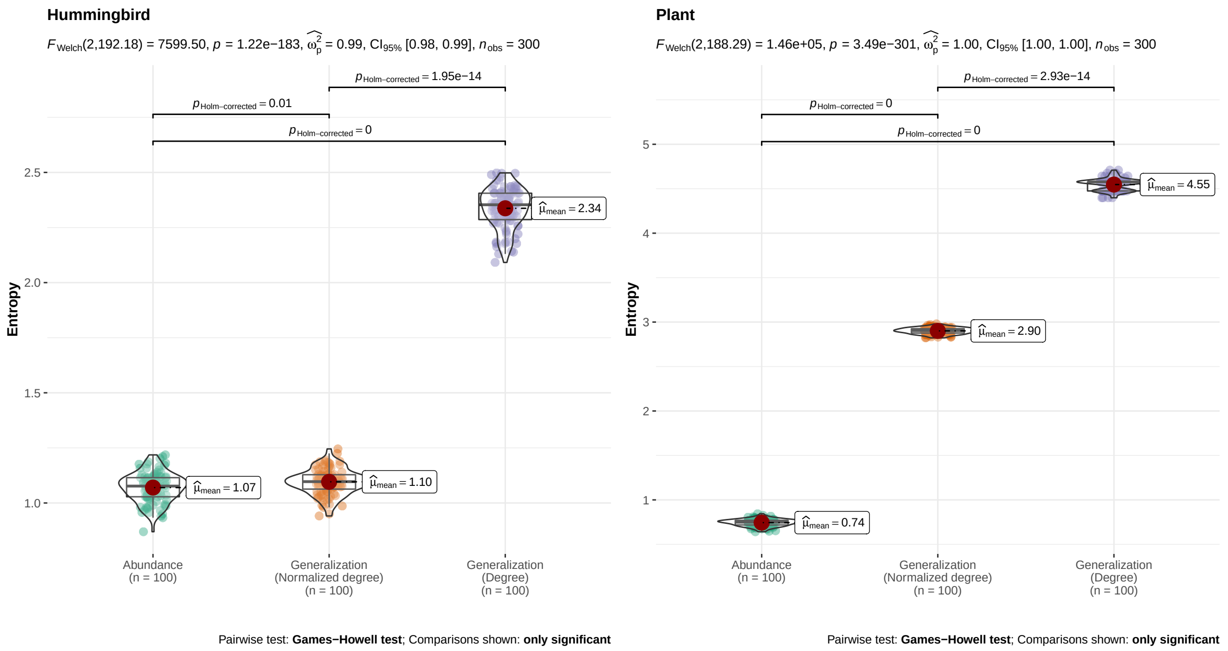

Fig. U. Estimation of empirical entropy in information-geometric inference. The x axis shows the two categories of models: one with abundance as the cause (i.e., predictor in the regression), while the other with generalism as the cause. The y axis shows entropy. The points are from 100 boostraps.

S8 Analysis of the dataset of reef fishes

Figure V in S1 Text confirms a positive correlation between species abundance and generalism in reef fishes [17].

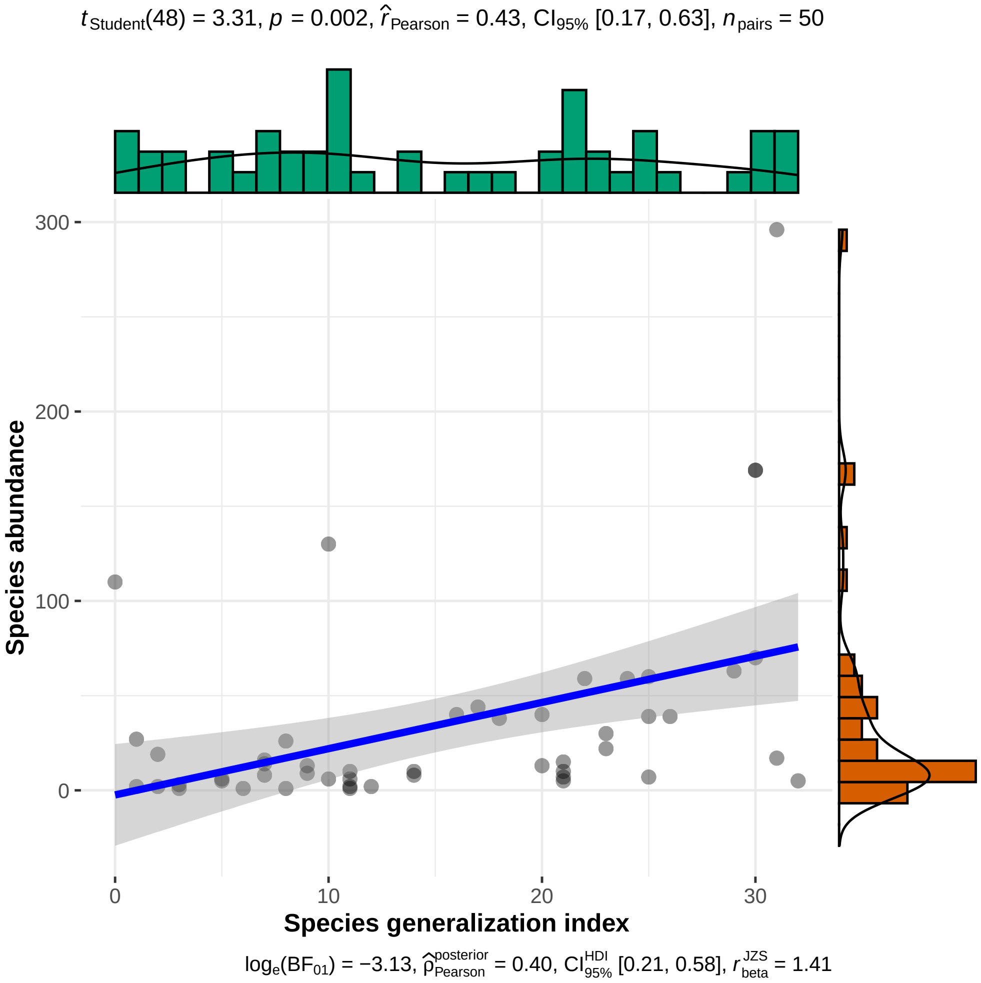

Fig. V. Correlation between species generalism and abundance in reef fishes.

The additive noise model shows that species generalism is more likely to be cause (Table A).

Table A: Additive noise model

	p value	adjusted R^2
generalism	0.23	0.28
Abundance	0.00	0.27

The information-geometric inference also shows that species generalism is more likely to be cause (Table B).

Table B: Empirical entropy of marginal distribution

	Entropy
generalism	1.62
Abundance	1.02

References

1. Abatzoglou, J. T., Dobrowski, S. Z., Parks, S. A. & Hegewisch, K. C. (2018). Terraclimate, a high-resolution global dataset of monthly climate and climatic water balance from 1958–2015. *Scientific data*, 5, 1–12.

2. AlAdwani, M. S. K. F. (2019). *Understanding the effects of functional responses in ecological dynamical systems.* Ph.D. thesis, Massachusetts Institute of Technology.

3. Azadkia, M. & Chatterjee, S. (2019). A simple measure of conditional dependence. *arXiv preprint arXiv:1910.12327.*

4. Azadkia, M., Chatterjee, S. & Matloff, N. (2021). *FOCI: Feature Ordering by Conditional Independence.* URL `https://CRAN.R-project.org/package=FOCI`. R package version 0.1.3.

5. Blüthgen, N., Menzel, F. & Blüthgen, N. (2006). Measuring specialization in species interaction networks. *BMC Ecology*, 6, 1–12.

6. Dormann, C. F., Gruber, B. & Fruend, J. (2008). Introducing the bipartite package: Analysing ecological networks. *R News*, 8, 8–11.

7. Dormann, C. F., Gruber, B. & Fruend, J. (2008). Introducing the bipartite package: Analysing ecological networks. *R News*, 8, 8–11.

8. Fort, H., Vázquez, D. P. & Lan, B. L. (2016). Abundance and generalisation in mutualistic networks: solving the chicken-and-egg dilemma. *Ecology Letters*, 19, 4–11.

9. Newman, M. E. (2006). Modularity and community structure in networks. *Proceedings of the national academy of sciences*, 103, 8577–8582.

10. Peterson, R. A. & Cavanaugh, J. E. (2019). Ordered quantile normalization: a semiparametric transformation built for the cross-validation era. *Journal of Applied Statistics*, 47, 2312–2327.

11. Pfister, N., Bühlmann, P., Schölkopf, B. & Peters, J. (2018). Kernel-based tests for joint independence. *Journal of the Royal Statistical Society Series B*, 80, 5–31.

12. Pfister, N. & Peters, J. (2019). *dHSIC: Independence Testing via Hilbert Schmidt Independence Criterion.* URL `https://CRAN.R-project.org/package=dHSIC`. R package version 2.1.

13. Simmons, B. I., Cirtwill, A. R., Baker, N. J., Wauchope, H. S., Dicks, L. V., Stouffer, D. B. & Sutherland, W. J. (2019). Motifs in bipartite ecological networks: uncovering indirect interactions. *Oikos*, 128, 154–170.

14. Simmons, B. I., Sweering, M. J., Schillinger, M., Dicks, L. V., Sutherland, W. J. & Di Clemente, R. (2019). bmotif: A package for motif analyses of bipartite networks. *Methods in Ecology and Evolution*, 10, 695–701.

15. Song, C., Rohr, R. P. & Saavedra, S. (2017). Why are some plant–pollinator networks more nested than others? *Journal of Animal Ecology*, 86, 1417–1424.

16. Song, C., Rohr, R. P. & Saavedra, S. (2019). Beware z-scores. *Journal of Animal Ecology*, 88, 808–809.

17. Stuart-Smith, R. D., Mellin, C., Bates, A. E. & Edgar, G. J. (2021). Habitat loss and range shifts contribute to ecological generalization among reef fishes. *Nature Ecology & Evolution*, 5, 656–662.

18. Wood, S. (2017). *Generalized Additive Models: An Introduction with R.* 2nd edn. Chapman and Hall/CRC.

19. Wood, S. N. (2011). Fast stable restricted maximum likelihood and marginal likelihood estimation of semiparametric generalized linear models. *Journal of the Royal Statistical Society: Series B (Statistical Methodology)*, 73, 3–36.
